# Allele Frequencies of 10 Autosomal STR Loci from Chakma and Tripura Tribal Populations in Bangladesh

**DOI:** 10.4061/2010/740152

**Published:** 2010-06-06

**Authors:** Ahmad Ferdous, Mohammad Eunus Ali, Shafiul Alam, Mahamud Hasan, Tania Hossain, Sharif Akhteruzzaman

**Affiliations:** ^1^National Forensic DNA Profiling Laboratory, Department of Forensic Medicine, Dhaka Medical College, Dhaka 1000, Bangladesh; ^2^Department of Biochemistry and Molecular Biology, University of Dhaka, Dhaka 1000, Bangladesh

## Abstract

Allele frequencies of ten autosomal STR loci, D3S1358, vWA, D16S539, D2S1338, D8S1179, D21S11, D18S51, D19S433, TH01, and FGA were investigated in Chakma and Tripura tribal populations of Bangladesh. In both the populations, all loci were in Hardy-Weinberg equilibrium except for FGA locus in Chakma and D21S11 in Tripura. All the loci were highly polymorphic in Chakma population with an observed heterozygosity (*Ho*) of >0.7 and moderately polymorphic in Tripura population (*Ho* > 0.6). However, both the population showed least polymorphism at TH01 locus (*Ho* < 0.6). A comparison between Chakma and Tripura population data revealed statistically significant differences in allele frequency distribution for most of the loci. A similar comparison with the mainstream Bengali population using previously published data from this lab also showed significant difference in allele frequency with these two tribal populations.

## 1. Introduction

Short tandem repeats (STRs) are highly polymorphic sequences of nucleotides, which are abundant in eukaryotic genome. They form approximately 3% of the total human genome and occur on average in every 10 000 nucleotides [1]. Due to their small dimension, low mutation, and high level of polymorphism, these markers are intensely used as important genetic markers for mapping studies, disease diagnosis, and human identity testing [2]. STRs remained the mainstays in most of the forensic laboratories in the world for the last two decades, as these markers provide high statistical capability of discrimination and individualization [3]. With the growing number of laboratories which use STR markers, more and more population data are reported from all over the world. 

In this study, we report the allele frequency data from Chakma and Tripura tribal population of Bangladesh. Besides the forensic uses, we find this study a very powerful adjunct for further regional and worldwide meta analysis.

## 2. Materials and Methods

### 2.1. Population

Liquid blood samples were collected from randomly selected 109 Chakma and 58 Tripura individuals in Bangladesh. There are four ethnic groups in Bangladesh; the Dravidian, proto-Australian, Mongolian, and Bengalis. The Bengalis are by far of all, constituting 98% of the population of the country. The Chakmas and Tripuras tribal groups along with Marmas, Garos, and Manipuris belong to the Mongoloid stock and are Sino-Tibetan in origin. They live in Chittagong Hill Tract, Khagrachari, and Bandarban districts along the eastern border of Bangladesh adjoining Myanmar and Indian states of Mizoram and Tripura. The Chittagong Hill Tracts host 13 main tribes of which Chakmas are largest single tribes in Bangladesh. According to the 2001 population census, there were about 300 000 Chakmas and 90 000 Tripuras. The Dravidian element of population is represented mainly by Oraons, a tribe of central India in origin. The Australoid group includes Khasias and Santals, mainly labourers in the tea garden at the Sylhet district.

### 2.2. DNA Extraction

DNA was extracted using the Chelex-100 method as described by Walsh et al. [4]. Extracted DNA was quantified by using NanoDrop-1000 (NanoDrop Technologies, Inc., Wilmington, DE 19810, USA).

### 2.3. PCR Amplification

Approximately 1-2 ng of template DNA was used for each PCR amplification process. Ten autosomal STR loci namely D3S1358, vWA, D16S539, D2S1338, D8S1179, D21S11, D18S51, D19S433, TH01, and FGA were coamplified using AmpF*l*STR SGM Plus PCR amplification kit (Applied Biosystems, Foster City, CA, USA). The PCR reaction was carried out in a GenAmp PCR System 2720 (Appliled Biosystems). Thermal cycling parameters were setup according to the manufacturer's protocol.

### 2.4. STR Typing

PCR-amplified fragments were separated and analyzed on ABI Prism 3100-*avant* Genetic Analyzer (Applied Biosystems) using POP-4 polymer and data collection software ver. 1.1. Data were sized using GeneScan Software version 3.7 and internal GeneScan-500 ROX size standard. Genotype of each locus was determined after comparison with allelic ladder using Genotyper software version 3.7 NT.

### 2.5. Analysis of Data

Allele frequencies at each locus and statistical parameters of forensic efficiency were calculated by using PowerStat Microsoft Excel Workbook template [5]. Possible divergence from Hardy-Weinberg equilibrium was evaluated by Fisher's exact test [6]. Population differentiation test using exact test was carried out using Arlequine 2.0 software [7].

### 2.6. Quality Control

Positive control DNA and allelic ladder provided in AmpF*l*STR SGM Plus PCR amplification kit (Applied Biosystems). Approximately 10% of samples from both the populations were regenotypes to ensure reproducibility and accuracy. All genotype results were in full concordance.

## 3. Results and Discussion

Allele frequencies of Chakma and Tripura tribal groups are shown in Tables 1 and 2, respectively. Forensic efficiency parameters for both the populations are summarized in Table 3. Population differentiation test per locus is summarized in Table 4. The combined probability of match (PM) for the 10 STR loci are 3.49 × 10^−12^ (1 in 2.86 × 10^11^) and 1.03 × 10^−11^ (1 in 9.65 × 10^10^), respectively, for Chakma and Tripura populations. The combined power of exclusion of paternity (PE) for the Chakma and Tripura were calculated to be 0.99997 and 0.9998, respectively. Significant deviation from Hardy-Weinberg equilibrium was observed for FGA locus in Chakma and D21S11 in Tripura population. Both the populations showed least polymorphism at TH01 locus (PIC <0.6). This may be due to inbreeding and lack of admixture, which is a characteristic feature of these populations. Although both the populations achieved combined probability of match (PM) in the order of 10^10^ to 10^11^, it would be reasonable to implement a “*θ* correction” while using this data in forensic casework, as recommended by the National Research Council (1996) [8] and by Foreman and Lambert [9]. Population differentiation test for each population pair per locus revealed significant difference in the allele frequency distribution for most of the loci (Table 4) between Chakma and Tripura populations. Both Chakma and Tripura populations also showed significant difference in the allele frequency distribution with the mainstream Bengali population when compared with previously published data from this lab [10]. Among the populations THO1 locus showed least differentiation whereas, highest differentiation was observed in D18S51 locus which was followed by D2S1338 locus (Table 4). 

The present study was undertaken from the standpoint of compiling own-population genetic database, owing to the fact that population and subpopulation differences at STR loci of forensic interest are growing up. In this context, we embarked on compiling the allele frequency database of two ethnolinguistic populations of Bangladesh. The obtained high combined PM and PE values demonstrated that STR loci employed in SGM Plus PCR amplification kit would be highly efficient for personal identification and paternity cases in spite of some population substructuring. Besides forensic benefits, we find this study very useful for regional and worldwide human population meta-analysis.

## Figures and Tables

**Table 1:** Allele frequency distribution of 10 autosomal STR loci in Chakma population (*n* = 109).

Allele	D3S1358	vWA	D16S539	D2S1338	D8S1179	D21S11	D18S51	D19S433	TH01	FGA
5	—	—	—	—	—	—	—	—	—	—
6	—	—	—	—	—	—	—	—	0.0412	—
7	—	—	—	—	—	—	—	—	0.2568	—
8	—	—	0.0366	—	—	—	—	—	0.0596	—
9	—	—	0.3073	—	—	—	—	—	0.5871	—
9.3	—	—	—	—	—	—	—	—	0.0137	—
10	—	—	0.1055	—	0.1284	—	—	—	0.0412	—
11	—	—	0.2752	—	0.0779	—	—	—	—	—
12	—	—	0.1559	—	0.0596	—	0.0779	0.0091	—	—
13	—	—	0.1100	—	0.1926	—	0.1422	0.1697	—	—
13.2	—	—	—	—	—	—	—	0.0504	—	—
14	0.0596	0.1055	0.0091	—	0.2981	—	0.1743	0.2889	—	—
14.2	—	—	—	—	—	—	—	0.1238	—	—
15	0.2431	0.0091	—	—	0.1146	—	0.1788	0.1651	—	0.0046
15.2	—	—	—	—	—	—	—	0.0688	—	—
16	0.4266	0.1651	—	0.0091	0.1192	—	0.1376	0.0458	—	—
16.2	—	—	—	—	—	—	—	0.0733	—	—
17	0.2201	0.3027	—	0.0183	—	—	0.0779	0.0045	—	0.0092
17.2	—	—	—	—	—	—	—	—	—	—
18	0.0458	0.2247	—	0.1559	—	—	0.0458	—	—	0.0092
19	0.0045	0.1651	—	0.2935	0.0091	—	0.0642	—	—	0.0688
20	—	0.0275	—	0.1192	—	—	0.0229	—	—	0.0688
21	—	—	—	0.0091	—	—	0.0550	—	—	0.1239
21.2	—	—	—	—	—	—	—	—	—	0.0092
22	—	—	—	0.0504	—	—	0.0091	—	—	0.1881
22.2	—	—	—	—	—	—	—	—	—	0.0046
23	—	—	—	0.1192	—	—	—	—	—	0.1147
23.2	—	—	—	—	—	—	—	—	—	0.0367
24	—	—	—	0.1605	—	—	0.0137	—	—	0.1835
24.2	—	—	—	—	—	—	—	—	—	0.0092
25	—	—	—	0.0596	—	—	—	—	—	0.1009
25.2	—	—	—	—	—	—	—	—	—	0.0046
26	—	—	—	0.0045	—	—	—	—	—	0.0413
26.2	—	—	—	—	—	—	—	—	—	0.0138
27	—	—	—	—	—	—	—	—	—	0.0092
28	—	—	—	—	—	0.0504	—	—	—	—
28.2	—	—	—	—	—	0.0091	—	—	—	—
29	—	—	—	—	—	0.2339	—	—	—	—
29.2	—	—	—	—	—	—	—	—	—	—
30	—	—	—	—	—	0.2155	—	—	—	—
30.2	—	—	—	—	—	0.0458	—	—	—	—
31	—	—	—	—	—	0.0550	—	—	—	—
31.2	—	—	—	—	—	0.0275	—	—	—	—
32	—	—	—	—	—	0.0091	—	—	—	—
32.2	—	—	—	—	—	0.2477	—	—	—	—
33	—	—	—	—	—	0.0045	—	—	—	—
33.2	—	—	—	—	—	0.0825	—	—	—	—
34	—	—	—	—	—	—	—	—	—	—
34.2	—	—	—	—	—	0.0183	—	—	—	—

**Table 2:** Allele frequency distribution of 10 autosomal STR loci in Tripura population (*n* = 58).

Allele	D3S1358	vWA	D16S539	D2S1338	D8S1179	D21S11	D18S51	D19S433	TH01	FGA
5	—	—	—	—	—	—	—	—	—	—
6	—	—	—	—	—	—	—	—	0.0603	—
7	—	—	—	—	—	—	—	—	0.2068	—
8	—	—	—	—	—	—	—	—	0.0431	—
9	—	—	0.2758	—	—	—	—	—	0.5948	—
9.3	—	—	—	—	—	—	—	—	0.0948	—
10	—	—	0.1724	—	0.1465	—	—	—	—	—
11	—	—	0.4137	—	0.0517	—	—	—	—	—
12	—	—	0.1034	—	0.1120	—	0.0517	0.0258	—	—
12.2	—	—	—	—	—	—	—	0.0086	—	—
13	—	—	0.0258	—	0.1551	—	0.1379	0.0948	—	—
13.2	—	—	—	—	—	—	—	0.0775	—	—
14	0.0172	0.0948	0.0086	—	0.2586	—	0.1724	0.3706	—	—
14.2	—	—	—	—	—	—	—	0.0689	—	—
15	0.2586	0.0172	—	—	0.1982	—	0.1637	0.1206	—	—
15.2	—	—	—	—	—	—	—	0.1896	—	—
16	0.4482	0.1896	—	—	0.0775	—	0.1637	—	—	—
16.2	—	—	—	—	—	—	—	0.0344	—	—
17	0.1724	0.2155	—	0.0689	—	—	0.0603	—	—	—
17.2	—	—	—	—	—	—	—	0.0086	—	—
18	0.1034	0.2413	—	0.1034	—	—	0.0948	—	—	0.0258
19	—	0.1551	—	0.2413	—	—	0.0775	—	—	0.0431
20	—	0.0517	—	0.1293	—	—	0.0603	—	—	0.1551
21	—	0.0344	—	0.0344	—	—	0.0086	—	—	0.0517
21.2	—	—	—	—	—	—	—	—	—	0.0344
22	—	—	—	0.0258	—	—	—	—	—	0.1206
22.2	—	—	—	—	—	—	—	—	—	0.0172
23	—	—	—	0.2155	—	—	0.0086	—	—	0.1810
23.2	—	—	—	—	—	—	—	—	—	0.0172
24	—	—	—	0.1293	—	—	—	—	—	0.1465
24.2	—	—	—	—	—	—	—	—	—	0.0172
25	—	—	—	0.0258	—	—	—	—	—	0.1465
25.2	—	—	—	—	—	—	—	—	—	—
26	—	—	—	0.0258	—	—	—	—	—	0.0258
26.2	—	—	—	—	—	—	—	—	—	—
27	—	—	—	—	—	0.0172	—	—	—	0.0172
28	—	—	—	—	—	0.0344	—	—	—	—
28.2	—	—	—	—	—	0.0258	—	—	—	—
29	—	—	—	—	—	0.1896	—	—	—	—
29.2	—	—	—	—	—	—	—	—	—	—
30	—	—	—	—	—	0.2931	—	—	—	—
30.2	—	—	—	—	—	0.0431	—	—	—	—
31	—	—	—	—	—	0.0517	—	—	—	—
31.2	—	—	—	—	—	0.0689	—	—	—	—
32	—	—	—	—	—	0.0086	—	—	—	—
32.2	—	—	—	—	—	0.2068	—	—	—	—
33	—	—	—	—	—	0.0086	—	—	—	—
33.2	—	—	—	—	—	0.0517	—	—	—	—
34	—	—	—	—	—	—	—	—	—	—
34.2	—	—	—	—	—	—	—	—	—	—

**Table 3:** Forensic parameters of 10 autosomal STR loci in Chakma and Tripura populations.

Chakma (*n* = 109)										
Allele	D3S1358	vWA	D16S539	D2S1338	D8S1179	D21S11	D18S51	D19S433	TH01	FGA

*Ho*	0.743	0.844	0.798	0.816	0.761	0.853	0.944	0.825	0.578	0.880
*He*	0.704	0.791	0.780	0.828	0.820	0.821	0.876	0.830	0.582	0.879
PM	0.145	0.086	0.089	0.062	0.056	0.059	0.039	0.055	0.224	0.032
PD	0.855	0.914	0.911	0.938	0.944	0.941	0.961	0.945	0.776	0.968
PIC	0.657	0.760	0.748	0.808	0.798	0.799	0.864	0.810	0.531	0.867
PE	0.498	0.683	0.596	0.630	0.530	0.701	0.888	0.648	0.265	0.756
TPI	1.946	3.206	2.477	2.725	2.096	3.406	9.083	2.868	1.185	4.192
*P*	0.631	0.821	0.991	0.393	0.577	0.239	0.060	0.180	0.661	0.001

Tripura (*n* = 58)										

Allele	D3S1358	vWA	D16S539	D2S1338	D8S1179	D21S11	D18S51	D19S433	TH01	FGA

*Ho*	0.603	0.862	0.689	0.827	0.896	0.758	0.896	0.793	0.551	0.810
*He*	0.691	0.822	0.711	0.843	0.826	0.821	0.872	0.790	0.588	0.877
PM	0.159	0.080	0.127	0.050	0.071	0.062	0.045	0.071	0.228	0.040
PD	0.841	0.920	0.873	0.950	0.929	0.938	0.955	0.929	0.772	0.960
PIC	0.642	0.798	0.664	0.825	0.804	0.800	0.859	0.767	0.547	0.865
PE	0.295	0.719	0.412	0.651	0.788	0.525	0.788	0.586	0.237	0.618
TPI	1.261	3.625	1.611	2.900	4.833	2.071	4.833	2.417	1.115	2.636
*P*	0.933	0.244	0.536	0.433	0.665	0.042	0.381	0.175	0.678	0.319

**Table 4:** Exact test of population differences based on allele frequency.

Population pair	D3S1358	vWA	D16S539	D2S1338	D8S1179	D21S11	D18S51	D19S433	TH01	FGA
Chakma-Tripura (*P*)	0.003	0.011	0.015	0.000	0.000	0.173	0.000	0.008	1.000	0.015
Bengali-Chakma (*P*)	0.000	0.001	0.024	0.000	0.036	0.009	0.000	0.003	1.000	0.055
Bengali-Tripura (*P*)	0.003	0.011	0.061	0.001	0.006	0.263	0.000	0.098	1.000	0.000

*P-*values <0.05 were considered significantly different.
